# Decrease in cognitive performance and increase of the neutrophil-to-lymphocyte and platelet-to-lymphocyte ratios with higher doses of antipsychotics in women with schizophrenia: a cross-sectional study

**DOI:** 10.1186/s12888-023-05050-x

**Published:** 2023-08-02

**Authors:** Ilgner Justa Frota, Alissandra Lima Barbosa de Oliveira, David Nunes De Lima, Carlos Winston Luz Costa Filho, Carlos Eduardo de Souza Menezes, Michelle Verde Ramo Soares, Adriano José Maia Chaves Filho, Deniele Bezerra Lós, Roberta Tavares de Araújo Moreira, Glautemberg de Almeida Viana, Eugênio de Moura Campos, Silvânia Maria Mendes Vasconcelos, Mary V. Seeman, Danielle S. Macêdo, Lia Lira Olivier Sanders

**Affiliations:** 1 Neuropsychopharmacology and Translational Psychiatry Laboratory, Drug Research and Development Center, Faculty of Medicine, Federal University of Ceará, Rua Coronel Nunes de Melo, 1000 - Rodolfo Teófilo, Fortaleza, CE Postal Code 60430-275 Brazil; 2 Department of Clinical Medicine, Faculty of Medicine, Federal University of Ceará, Fortaleza, Brazil; 3 Department of Medical Education, Faculdade Paraíso, Araripina, Brazil; 4 Department of Psychology, Christus University Center, Fortaleza, Brazil; 5 Laboratory of Clinical and Toxicological Analysis, Department of Pharmacy, Federal University of Ceará, Fortaleza, Brazil; 6 Department of Psychiatry, University of Toronto, Toronto, Canada

**Keywords:** Neuropsychological tests, Antipsychotics, Inflammation, Schizophrenia, Neutrophil-to-lymphocyte ratio, Executive function

## Abstract

**Background:**

We explored the relationship between symptoms, cognitive performance, neutrophil-to-lymphocyte ratio (NLR), monocyte-to-lymphocyte ratio (MLR), and platelet-to-lymphocyte ratio (PLR) (three markers of inflammation), and antipsychotic dose (in chlorpromazine units) in male and female patients with schizophrenia.

**Methods:**

We conducted a cross-sectional analysis in patients with schizophrenia of the complete blood count and the results of neuropsychological testing, using the Welch t-test to compare groups and the Pearson test for correlations.

**Results:**

We found that the NLR and the PLR are higher among women with schizophrenia when compared with men. In women, the NLR and the PLR correlate positively with antipsychotic drug dose and inversely with a working memory test (Direct Digit Span). Higher doses of antipsychotics are associated with worse working and semantic memory and mental flexibility in the women in our sample.

**Conclusion:**

Higher doses of antipsychotics were associated with worse working and semantic memory and mental flexibility in women with schizophrenia. No such correlations were present in men, suggesting that, in female patients, cognitive performance deteriorates as the antipsychotic dose is increased, a finding that could be mediated by inflammatory mechanisms, given the demonstrated relationship to biomarkers of inflammation – e.g., the NLR and the PLR.

**Trial registration:**

NCT03788759 (ClinicalTrials.gov).

## Background

Schizophrenia is widely considered to be an inflammatory disease associated with chronic neural and systemic inflammation. Pro-inflammatory cytokines play a role in neurogenesis [1], and treatment with antipsychotic medications may, to some extent, work by inducing anti-inflammatory and antioxidant effects [2]. Therefore, identifying neuroinflammatory biomarkers in schizophrenia may be useful in diagnosis, in determining optimal antipsychotic dose, and in tracking treatment efficacy.

The clinical impact of antipsychotics differs depending on patient sex [3]. Cardiovascular death, for instance, to which men are generally more susceptible than women, is disproportionately increased in women by antipsychotic treatment [4]. Estrogen has direct antipsychotic effects at brain receptor sites and modulates the metabolism of some (not all) antipsychotics [3, 5]. Due, at least in part, to these mechanisms, women with schizophrenia of childbearing age need lower doses of antipsychotics than men [5]. As current dosing guidelines make no sex-specific recommendations, leaving women open to cumulative adverse effects, biomarkers indicative of treatment response need urgently to be developed.

The neutrophil-to-lymphocyte ratio (NLR) is a reasonably good marker of systemic inflammation that correlates with cardiac events and mortality related to many cardiovascular diseases [6]. It has substantial relevance as a biomarker due to its simplicity and ready availability. Requiring only an inexpensive and relatively unintrusive Complete Blood Count (CBC), this accessible biomarker can be easily measured by dividing the absolute neutrophil count by the lymphocyte count [7]. The NLR links the innate immune response, represented by neutrophils, to adaptive immunity, represented by lymphocytes [8]. It is, thus, a marker of pathogenetic inflammation, which plays an important part not only in schizophrenia [7] but also in several medical conditions – e.g., cardiovascular disease [6].

NLR is increased relative to the general population in both first-episode psychosis and chronic forms of schizophrenia [9], but, especially in the latter, antipsychotic effects constitute a confounding factor. The NLR increases with the severity of psychopathology in schizophrenia and decreases with antipsychotic administration [10]. An elevated NLR is also significantly associated with an increased risk of mild cognitive impairment [11]. While we have found no studies on the relationship between the NLR and cognition in schizophrenia, this marker has been shown to be inversely related to cognitive test scores in bipolar disorder [12]. Concerning sex/gender differences, most studies report better overall cognitive function in women with schizophrenia than in men [13]. There is a positive relationship among cognition, sex hormones, and inflammatory markers [14]. There are reports of further sex/gender differences in schizophrenia in: neuroanatomical characteristics [13], clinical manifestations, and disease progression [15].

The monocyte-to-lymphocyte ratio (MLR) and the platelet-to-lymphocyte ratio (PLR) are two other accessible blood biomarkers. Though they are less well-studied than the NLR, some reports suggest that they also may reflect inflammatory/anti-inflammatory processes in schizophrenia and its treatment. All three markers show higher levels in schizophrenia during relapse than during remission [16]. In addition, high raw monocyte counts have also been correlated with the presence of schizophrenia [17]. Furthermore, some of these biomarkers have been observed to differ according to sex [18].

We conducted a previous clinical trial with schizophrenia patients [19] in which we analyzed the baseline Complete Blood Count but did not examine NLR, MLR, or PLR [19]. Because there is a relative dearth of research on the role of these markers in schizophrenia [7], particularly in relation to biological sex, our aim was to conduct a cross-sectional, posthoc analysis of the baseline data of the earlier clinical trial [19], looking specifically at the NLR, MLR, and PLR. We hypothesized that, despite the limitations intrinsic to such a method, we could provide a solid starting point for high-quality analyses of the variability by sex of these biomarkers in schizophrenia. We explored the relationship among the following: NLR, MLR, PLR, symptomatology, cognitive performance, and antipsychotic dose in male and female patients with schizophrenia. Our aim was to test the hypothesis that the three inflammatory markers we had selected predicted cognitive defects in schizophrenia in a sex-specific manner.

## Methods

We began by analyzing the baseline data of our previous clinical schizophrenia trial, the details of which are described in clinical protocol NCT03788759 (ClinicalTrials.gov), and results published in reference [19]. That trial examined the effect of alpha-lipoic acid on several clinical and laboratory measures, the baseline values of which we reanalyze here. For the present study, we look at three parameters we had not considered previously: the NLR, MLR, and PLR. All three are calculated as simple ratios of the relevant peripheral blood cell count (neutrophil, monocyte, or platelet, respectively) over the lymphocyte counts. These are markers of inflammation, which we measure against cognitive performance and compare in men versus women, taking antipsychotic dose into account.

The sample consisted of 35 patients (12 women and 23 men) between 18 and 60 and diagnosed with schizophrenia, according to the Diagnostic and Statistical Manual of Mental Disorders, Fifth Edition [20]. In the original clinical trial, the required sample size determination was 19 participants per group (β = 0.10; α = 0.05) [19], We, thus, recruited 25 patients per group to cover attrition losses; however, losses were larger than expected (27% dropout rate), which left us with an undersized sample.

All patients had been on stable antipsychotic doses for at least one year prior to the study. All were in stable condition. Exclusion criteria were: history of epilepsy or cerebral tumor; current valproic acid use; severe gastric, hepatic, renal, or cardiac disease; alcoholic psychosis; drug dependence, pregnancy, or lactation; co-morbid psychiatric diagnoses. All patients provided free and informed consent according to established ethical guidelines. Participants were verbally informed of their rights, the research protocol and purpose were thoroughly explained, and they provided written consent. In some cases, where decisional capacity was in doubt, the patient and a legal guardian signed consent. The research ethics committee of the Walter Cantídio University Hospital (CEP/HUWC) approved the study protocol (92598718.1.0000.5054).

We calculated the baseline antipsychotic dose in chlorpromazine equivalent units using a conversion table [21] and derived the NLR, MLR, and PLR from the CBC. We analyzed the relationship of these parameters to patients’ neuropsychological baseline performance on the following standardized tests: Trail Making Test [22], Block Corsi Test [23], Digit Span Task [24], Category (Animal) Fluency, Controlled Oral Word Association Test [25], Controlled Oral Word Association (FAS) Test [26], Rey Auditory Verbal Learning Test (RAVLT) [27], and Stroop Color and Word Test [28]. These tests measure the following cognitive functions, respectively: visual search, motor velocity, and mental flexibility (Trail Making); visuo-spatial working memory (Corsi); verbal working memory (Digit Span); semantic memory organization and grouping capacity (Category Fluency); verbal fluency (Controlled Oral Word Association); memory (Rey); and working memory, selective attention and visual search (Stroop).

After testing the relevant data sets for normality with the Shapiro-Wilk test, we checked for correlations using the Pearson correlation coefficient. For comparison between groups, we used the Welch *t*-test, as some of the subgroups had different variances due to outliers, thus violating one of the Student’s t-test assumptions. We used the significance cutoff *p*-value of 0.05 (two-tailed). The IBM Statistical Package for the Social Sciences, version 20, was used in the analyses.

## Results

A total of 35 patients participated in the study: twelve women and 23 men. The NLR was available for all the women and 22 men. Eleven women and 22 men took part in the neuropsychological testing. Demographic and baseline anthropometric data (sex, age, education, years of psychiatric illness, number of previous psychiatric hospitalizations, and body mass index) are described below (Table 1). As shown in our previous publication, there were no significant differences between men and women in demographic and clinical data [19].Most of the patients were on olanzapine (34.3%), haloperidol (34.3%), or risperidone (31.4%), while only 5.7% were on Clozapine. These numbers add up to over a 100%, as 31.4% of the patients used more than one antipsychotic. Detailed information on the antipsychotic regimen of every patient is available in our previous publication [19].

**Table 1:** Group comparison between sexes on demographic and clinical data, using the two-tailed Welch t-test (Mean ± SD for parametric data; Median (First quartile; Third quartile) for non-parametric data)

Parameter	Male (n = 22)	Female (n = 11)	*t* value
Age (years)	37.48 ± 7.19	38.33 ± 9.76	0.79
Antipsychotic daily dose (CPZ units)	211.13 ± 83.62	218.00 ± 120.97	0.86
Body Mass Index (BMI) (kg/m²)	29.57 ± 5.70	27.99 ± 3.88	0.34
Brief Psychiatric Rating Scale (BPRS)	10 (9; 16)	14 (10.25; 24)	0.10
Education (years)	11 (9; 12)	10 (6.5; 12)	0.57
Duration of disorder (years)	15 (6; 21)	15.5 (7.25; 20.75)	0.91
Number of hospitalizations	1 (0; 3)	1 (0; 2.75)	0.48

* Statistically significant (p < 0.05)

**Fig. 1:**
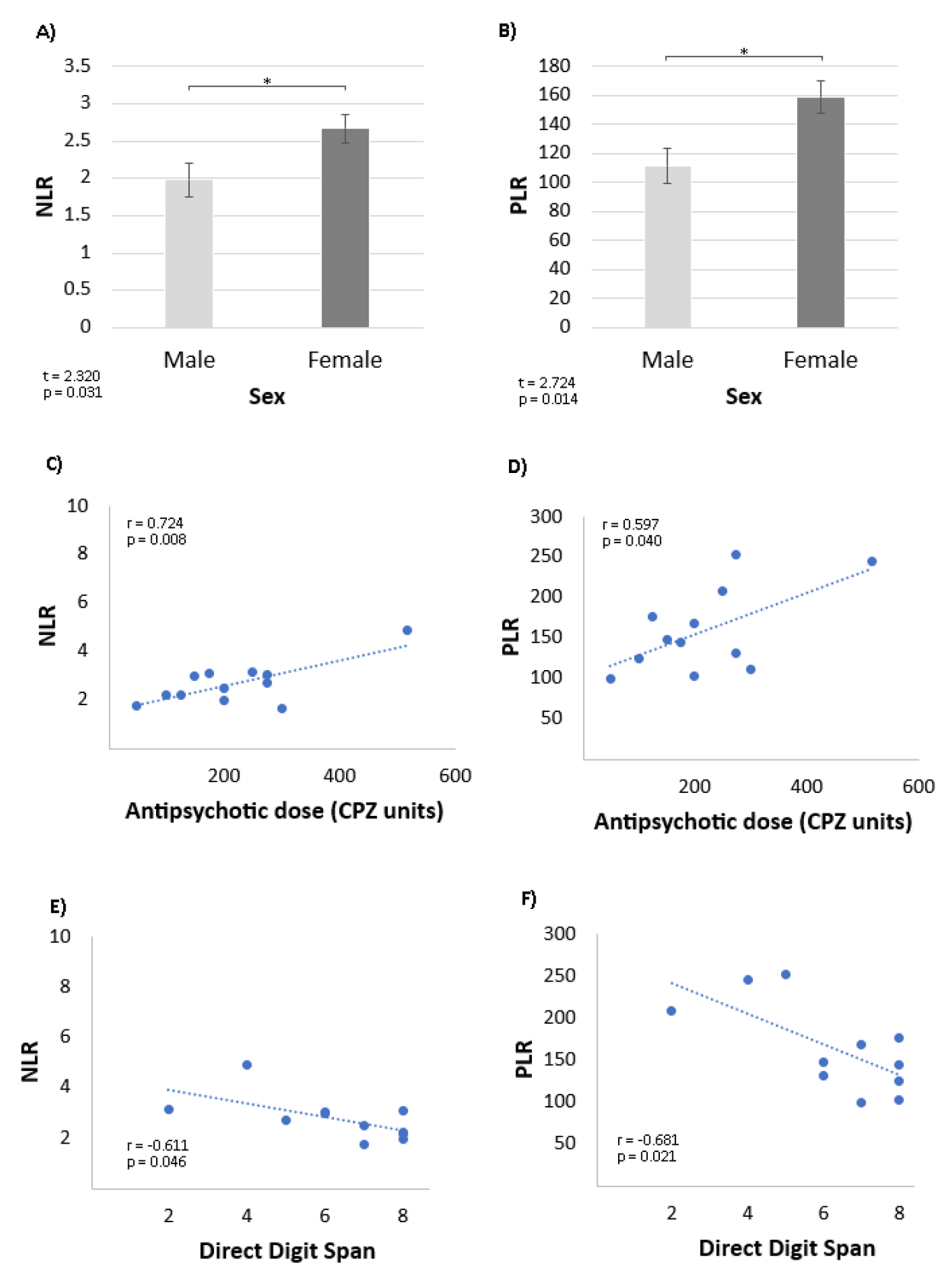
NLR and PLR in Each Sex and their Relationship to Antipsychotic Dose and Working Memory in Women. **A**) Neutrophil-to-lymphocyte Ratio (NLR) in each sex (error bars indicate SEM, the asterisk indicates p < 0.05). **B**) Platelet-to-lymphocyte Ratio (PLR) in each sex (error bars indicate SEM, the asterisk indicates p < 0.05). C) Correlation between NLR and daily antipsychotic dose (in Chlorpromazine-equivalent, CPZ units) in women (n = 12). (**D**) Correlation between PLR and daily antipsychotic dose (in Chlorpromazine-equivalent, CPZ units) in women (n = 12). (**E**) Relationship between the Direct Digit Span Task and NLR in women (n = 11). (**F**) Relationship between the Direct Digit Span Task and PLR in women (n = 11)

Female patients had a significantly higher NLR than male patients (Fig. 1a). We observed the same pattern with the PLR (Fig. 1b). However, no significant MLR difference was found between the sexes (t = 0.04; p = 0.97); thus, we did not evaluate this marker any further, as it cannot confirm or disconfirm our hypothesis. We found a positive correlation between the NLR and the daily antipsychotic dose in chlorpromazine equivalents in the female group (r = 0.724, Fig. 1c), indicating an increased imbalance between inflammation and immune response as the antipsychotic dose increases. We found the same pattern with the PLR (r = 0.597, Fig. 1d). Furthermore, the NLR and the PLR were inversely related to the direct Digit Span task performance in women but not in men (Fig. 1e and f), suggesting that, as inflammation increases, verbal working memory deteriorates. Among male patients, we found no meaningful correlations among any of these variables. We also found no significant correlations between the BPRS score and the NLR in either women (r = 0.34; p = 0.28), or men (r = -0.08; p = 0.36), or the total sample (r = 0.21; p = 0.22). The same holds true for the PLR.

**Fig. 2:**
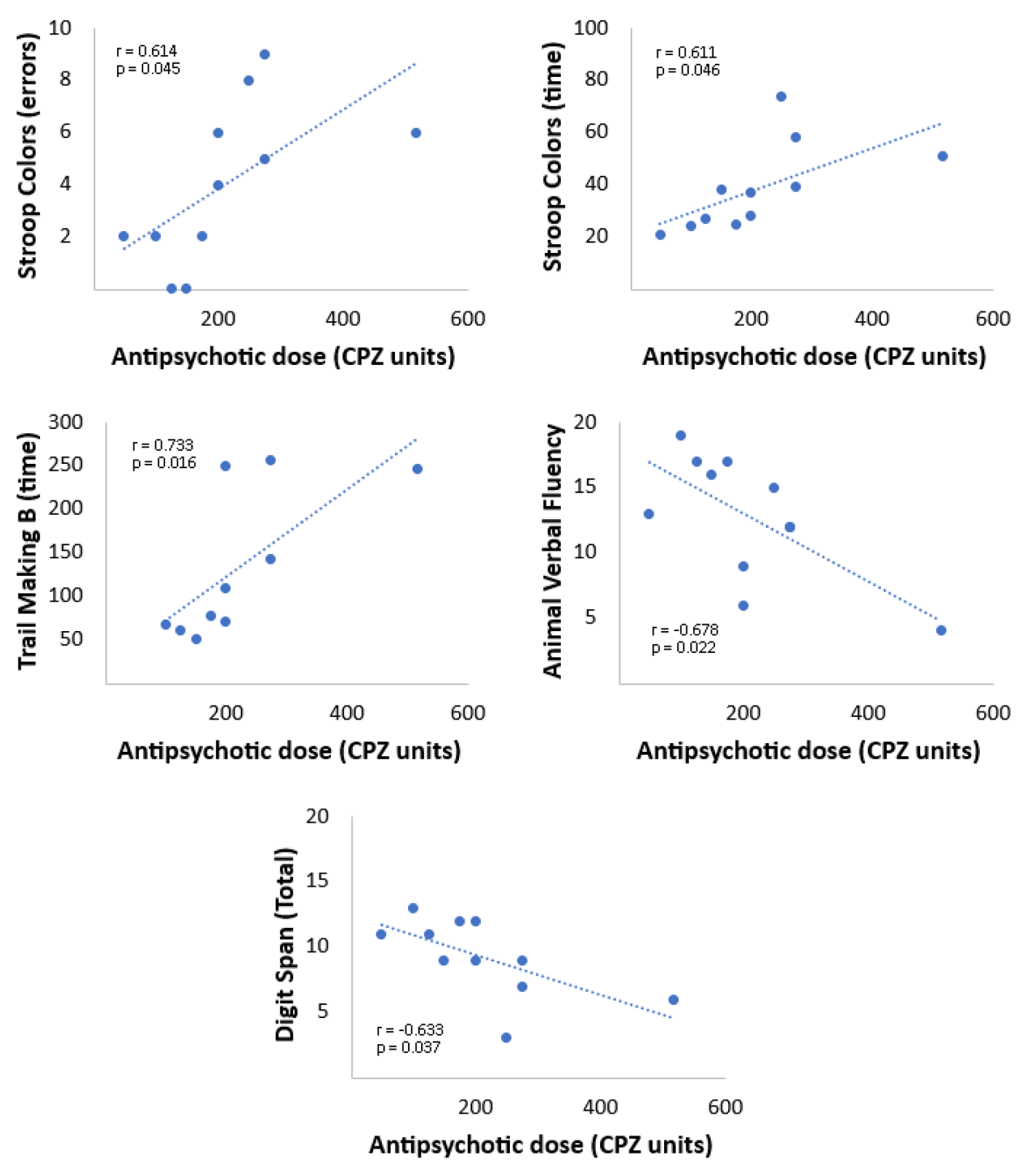
Correlation between Neuropsychological Tests and Antipsychotic Dose in Women. Correlation between neuropsychological tests (Digit Span task, Animal Verbal Fluency test, time taken in the Trail Making B test, and time and errors in the Stroop Colors test) and antipsychotic dose in CPZ units of females (n = 11)

Among women, there were significant correlations between the daily antipsychotic dose (in CPZ units) and several cognitive indicators (Table 2; Fig. 2). We found inverse relationships between the antipsychotic dose and the Digit Span and Animal Verbal Fluency tasks, which indicates a worsening of working memory and semantic memory, respectively, as the amount of antipsychotics increases. Further, there was a direct relationship between antipsychotic dose and the Trail Making B task response time and Stroop Colors task (total test response time and total test errors). Thus, as the drug dose increases, performance in these tests takes longer to complete, and, in the case of the Stroop Colors task, more errors are made.

**Table 2:** Correlation between neuropsychological tests and antipsychotic dose (CPZ units) in females

Test name	Task measured	Patients	Pearson correlation	*P* value
Digit Span Task	Correct sequence	11	-0.633	0.037*
Corsi Block Test	Correct sequence	11	-0.431	0.185
FAS Fluency Test	Words in one minute	11	-0.344	0.300
Animal Verbal Fluency Test	Animals in one minute	11	-0.678	0.022*
Trail Making A	Time in seconds	10	0.411	0.238
	Errors	10	0.263	0.462
Trail Making B	Time in seconds	10	0.733	0.016*
	Errors	10	0.392	0.263
Stroop Rectangles	Time in seconds	11	0.526	0.097
	Errors	11	0.106	0.757
Stroop Words	Time in seconds	11	0.571	0.067
	Errors	11	0.478	0.137
Stroop Colors	Time in seconds	11	0.611	0.046*
	Errors	11	0.614	0.045*
Rey 1–5	Correctness	11	0.015	0.964
Rey 6	Correctness	11	-0.283	0.399
Rey 7	Correctness	11	-0.151	0.658

* Statistically significant (p < 0.05)

There were no further significant correlations between antipsychotic dose, NLR or PLR and other cognitive tests in women – i.e., the Corsi Block test, the COWA (FAS) test, the Rey series of tests, and other measurements and varieties of the Trail Making and Stroop tests (Table 2). Additionally, we found no relationship between antipsychotic dose and cognitive performance in men, and we did not find any significant cognitive difference between the sexes (Table 3).

**Table 3:** Group comparison between sexes on neuropsychological tests, using the two-tailed Welch t-test (Mean ± SD).

Test name	Task measured	Male (n = 22)	Female (n = 11)	*t* value
Digit Span Task	Correct sequence	10.36 ± 3.74	9.27 ± 3.00	0.37
Corsi Block Test	Correct sequence	11.18 ± 3.23	9.82 ± 3.40	0.28
FAS Fluency Test	Words in one minute	16.00 ± 12.67	17.45 ± 12.82	0.76
Animal Verbal Fluency Test	Animals in one minute	13.82 ± 3.98	12.73 ± 4.78	0.52
Trail Making A	Time in seconds	56.91 ± 27.12	47.50 ± 25.89	0.36
	Errors	1.55 ± 2.20	2.30 ± 3.37	0.53
Trail Making B	Time in seconds	127.59 ± 66.05	133.00 ± 85.36	0.86
	Errors	7.82 ± 7.06	6.60 ± 6.62	0.64
Stroop Rectangles	Time in seconds	30.59 ± 21.98	24.36 ± 12.90	0.31
	Errors	1.23 ± 3.07	0.27 ± 0.90	0.19
Stroop Words	Time in seconds	28.50 ± 15.11	27.91 ± 14.54	0.91
	Errors	1.68 ± 2.97	1.09 ± 1.64	0.47
Stroop Colors	Time in seconds	39.86 ± 24.32	38.36 ± 16.55	0.84
	Errors	3.50 ± 3.56	4.00 ± 3.07	0.68
Rey 1–5	Correctness	30.05 ± 11.61	26.00 ± 12.79	0.39
Rey 6	Correctness	6.09 ± 2.94	6.82 ± 1.89	0.40
Rey 7	Correctness	6.27 ± 2.93	7.00 ± 2.32	0.45

* Statistically significant (p < 0.05)

## Discussion

We found a higher NLR and a higher PLR in women with schizophrenia compared to men. These inflammatory markers were also inversely related to working memory in women. Moreover, higher doses of antipsychotics worsened working and semantic memory and mental flexibility in the women in our sample. No such correlations were present in men, suggesting that, as antipsychotic doses increase, women show deteriorating cognitive performance and an imbalance between inflammation and immunity.

The cognitive areas most affected by antipsychotics were verbal working memory (as evaluated by the Digit Span task) and semantic memory (measured with the Animal Verbal Fluency test). Working memory is known to correlate with IQ [29], and poor semantic memory has been associated with the symptom of alogia [30], often seen in long-term schizophrenia. The Trail Making B test evaluates visual search, motor speed, and mental flexibility [31]. The same applies to the Stroop test, which measures cognitive inhibition (which can be related to increased impulsivity) and working memory (selective focus) [32]. These tests measure skills relevant to everyday function in the real world and, if our results are confirmed, would underscore the need to adjust antipsychotic treatment dose to a patient’s sex.

Many sex differences have been reported in schizophrenia [33], with controversy as to their origin, the question being being whether they result from biological or secondary environmental causes [34]. The results of this study research indicate a potential neurobiological basis for these differences.

As the NLR and PLR correlate positively with the chlorpromazine equivalent dose of antipsychotics, it appears that female patients are more sensitive than males to the effects of these drugs, perhaps because some estrogen receptors (such as the G Protein-Coupled Estrogen Receptor 1) mediate anti-inflammatory effects and are under-expressed in individuals with schizophrenia [35]. Neuroinflammation as a cause of this disorder and anti-inflammatory therapies are very active current areas of research in schizophrenia [36], as are estrogen-mediated sex differences in onset age, clinical presentation, and response to treatment [37].

The NLR has been independently associated with the severity of psychopathology in schizophrenia; it decreases after antipsychotic administration [10]. While the lack of correlation with symptoms in men is compatible with that finding since the patients were all medicated and had achieved symptom stability, our results suggest that women respond differently. Sex-specific reactions to similar antipsychotic doses could explain these surprising results [3]. In other words, women, who, on average, run a more favorable course of schizophrenia than men, at least during their reproductive years, should be treated with lower antipsychotic doses. Many women receive needlessly high doses, as existing dosing studies have been conducted chiefly in men [38]. It is known that antipsychotics can cause inflammatory damage [39] when their doses are too high for the individual being treated. The positive correlation between antipsychotic dose and the NLR and PLR suggests an increased side-effect burden in women. Simultaneously, oxidative stress from an overly high medication dose can result in impaired cognition.

Our study has limitations. It is a secondary analysis of a small subgroup of patients, making it underpowered for the purposes of this study, so that Type I and Type II error cannot be excluded. Furthermore, there are many inflammatory biomarkers that we did not evaluate, which is a potential source of bias. Additionally, we have not addressed the many sources of potential acute or chronic inflammation in patients nor checked for use of anti-inflammatory drugs, all potential confounders of our results. Furthermore, ours is a cross-sectional, non-interventional study with no healthy control group, which contributes to possible biases. However, even though our sample was small, we had enough women in the study to find between-group differences. We consider these preliminary findings a relevant starting point for future studies purposely designed from the ground up to evaluate sex differences in the relationship between cognition, antipsychotics, and inflammatory markers in schizophrenia.

## Conclusion

This study found a higher NLR and a higher PLR among women with schizophrenia when compared to men. In female patients, the NLR and the PLR correlated positively with the antipsychotic dose, which was negatively associated with the results of several cognitive performance tests. These findings add to the emerging understanding of schizophrenia as a neuroinflammatory disorder modulated by sex hormones. We hope our results foster future investigations of sex-specific distinctions, such as antipsychotic intolerance and its cognitive implications, and the role of the NLR and the PLR as relevant biomarkers for the diagnosis of this disorder and for tracking treatment efficacy.

## Data Availability

The datasets used and analyzed during the current study are available from the corresponding author on reasonable request.

## List of abbreviations

BMI: Body Mass Index
BPRS: Brief Psychiatric Rating Scale
CBC: Complete Blood Count
COWA: Controlled Oral Word Association
CPZ: Chlorpromazine
IQ: Intelligence Quotient
MLR: Monocyte-to-Lymphocyte Ratio
NLR: Neutrophil-to-Lymphocyte Ratio
PLR: Platelet-to-Lymphocyte Ratio
RAVLT: Rey Auditory Verbal Learning Test
SEM: Standard Error of the Mean
